# Chemopreventive Strategies for Inflammation-Related Carcinogenesis: Current Status and Future Direction

**DOI:** 10.3390/ijms18040867

**Published:** 2017-04-19

**Authors:** Yusuke Kanda, Mitsuhiko Osaki, Futoshi Okada

**Affiliations:** 1 Division of Pathological Biochemistry, Tottori University Faculty of Medicine, Yonago, Tottori 683-8503, Japan; kanda@med.tottori-u.ac.jp (Y.K.); osamitsu@med.tottori-u.ac.jp (M.O.); 2 Chromosome Engineering Research Center, Tottori University, Yonago, Tottori 683-8503, Japan

**Keywords:** inflammation-related carcinogenesis, chronic inflammation, chemoprevention

## Abstract

A sustained and chronically-inflamed environment is characterized by the presence of heterogeneous inflammatory cellular components, including neutrophils, macrophages, lymphocytes and fibroblasts. These infiltrated cells produce growth stimulating mediators (inflammatory cytokines and growth factors), chemotactic factors (chemokines) and genotoxic substances (reactive oxygen species and nitrogen oxide) and induce DNA damage and methylation. Therefore, chronic inflammation serves as an intrinsic niche for carcinogenesis and tumor progression. In this article, we summarize the up-to-date findings regarding definitive/possible causes and mechanisms of inflammation-related carcinogenesis derived from experimental and clinical studies. We also propose 10 strategies, as well as candidate agents for the prevention of inflammation-related carcinogenesis.

## 1. Introduction

In 1863, Rudolf Virchow hypothesized that cancers occurred at sites of chronic inflammation [1]. This hypothesis has been confirmed by epidemiological and experimental pathological studies. Parkin showed that infection-related inflammation contributed to approximately 20% of all cancer cases worldwide [2]. Inflammation-inducible factors, such as air pollution, foreign bodies and ultraviolet radiation, are also associated with carcinogenesis [3].

Since chronic inflammation is associated with more than one-fifth of cancer incidence, there is an urgent need to explore chemopreventive agents against inflammation-related carcinogenesis. Before clinical trials of such agents are initiated, it is necessary to understand the pathogenesis of inflammation-related carcinogenesis by using animal models [4]. For example, rodent models for *Helicobacter pylori* and inflammatory bowel disease, which are the major causes of human gastric and colon cancers, respectively, have been developed to elucidate the underlying pathogenic mechanisms [4,5]. Epidemiological studies have shown that chronic inflammation predisposes individuals to various cancers, including cancer of the gastrointestinal tract [6]. Therefore, the use of agents targeted against inflammatory mediators might be a promising approach to prevent various types of inflammation-related cancers. To date, food products, natural compounds and synthetic low-molecular-weight compounds have been shown to suppress inflammation-related carcinogenesis. In this review, we summarize the mechanisms of inflammation-induced carcinogenesis by classifying the mechanisms of action of chemopreventive agents, and we propose 10 strategies for the prevention of carcinogenesis.

## 2. Causes of Inflammation-Related Carcinogenesis

The International Agency for Research on Cancer (IARC), through its IARC Monographs Programme, has performed carcinogenic hazard assessment of agents in humans based on experimental and clinical reports [7]. In this assessment, agents are classified into five groups (Group 1, 2A, 2B, 3 and 4). Group 1 carcinogens are those that are definitely carcinogenic to humans (Table 1). Table 1 also summarizes presumed carcinogenic agents classified into Group 2A to 3, as well as other previously-reported presumed carcinogenic agents not included in the IARC study.

**Figure 1:**
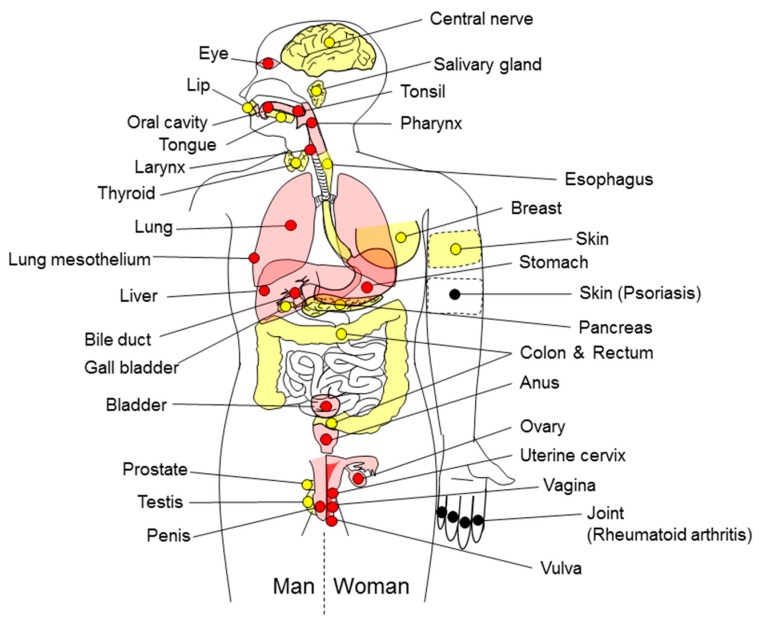
Organs/tissues involved in inflammation-related cancers. The organs/tissues with inflammation induced by definitely carcinogenic agents (red circles) or by presumed carcinogenic agents (yellow circles) are sensitive to cancer development. Skin (psoriasis) and joint (rheumatoid arthritis), indicated by black circles, are resistant to inflammation-related carcinogenesis.

**Figure 2:**
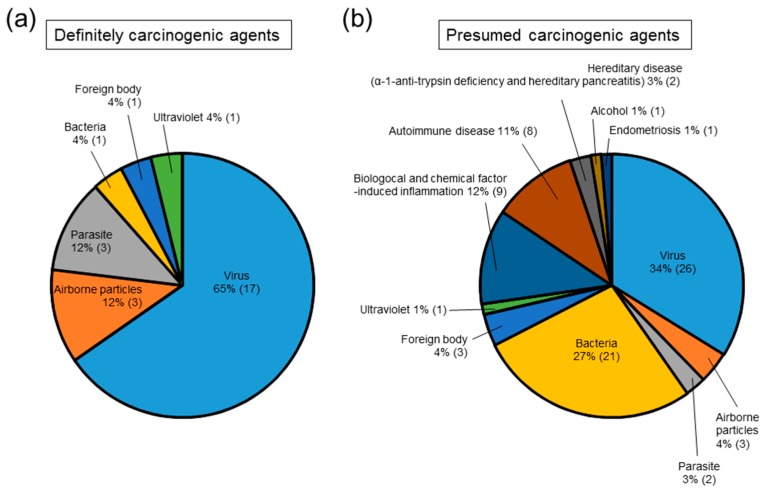
Causes of inflammation-related carcinogenesis. The proportion of definitely carcinogenic causes (**a**) or presumed carcinogenic causes (**b**) attributed to inflammation was derived from Table 1.

Chronic inflammation increases the risk of human cancers of almost all organs/tissues (Figure 1); however, some chronic inflammatory conditions (e.g., psoriasis and rheumatoid arthritis) are not associated with cancers. Figure 2a,b shows infection by viruses, bacteria and parasites as a percentage of all of the causes of inflammation-related cancers; this percentage is 81% for definitely carcinogenic agents and 64% for presumed carcinogenic agents. Readers should refer to other review articles for comprehensive information regarding viral, bacterial or parasitic infection-induced cancers [68,69,70]. It has recently been realized that inhalation of airborne particles (foreign body) is a novel cause of cancer. Here, we focus on this new cause of cancer, i.e., foreign body-induced carcinogenesis.

### Inhaled Foreign Body-Induced Carcinogenesis

A well-known carcinogenic foreign body is inhaled asbestos fibers, which are associated with mesothelioma and lung cancer (Table 1). The word “asbestos” is of Greek origin, being derived from “a”, meaning “not”, and “sbestos”, meaning “extinguishable”. Indeed, macrophages cannot remove the non-digestible asbestos fibers that lead to chronic inflammation [71].

There are three possible mechanisms for asbestos-induced carcinogenesis: (i) through the phenomenon of frustrated phagocytosis in which macrophages fail to phagocytose the long asbestos fibers and die with a massive release of reactive oxygen species (ROS) and pro-inflammatory cytokines that further induce chronic inflammation [72,73,74]; (ii) through asbestos-associated hemoglobin iron production of ROS via the Fenton reaction. This ROS damages DNA and stimulates the proliferation of alveolar epithelial cells and mesothelial cells [75]; and (iii) through asbestos induction of DNA double-stranded breaks in mesothelial cells, which leads to the promotion of genomic instability [73].

There was a general warning in 1973 that inhalation of asbestos causes lung cancer, gastrointestinal tract cancer and mesotheliomas [71]. The use of asbestos has since been banned in most developed countries; however, China and India still permit its usage [73]. Considering the latent period of mesothelioma (20 to 40 years after the first exposure to asbestos), its incidence is expected to increase further in the countries in which the peak of asbestos use was reached after the 1970s [71].

Not only manufactured products such as asbestos, but also airborne particles induce cancer. PM2.5 (particles with a diameter of 2.5 μm or less) can penetrate deeply into the lung, irritate and corrode the alveolar wall and lead to neutrophil infiltration [76]. Additionally, such gaseous particles were shown to decrease pulmonary function in schoolchildren [77]. This effect was caused by their induction of the overproduction of interleukin (IL)-8, an inflammatory cytokine [78]. Asian dust (AD) originates in China and transports a large amount of particulate matter to East Asian countries, such as Korea and Japan. In these countries, exposure to AD is associated with a decrease in the pulmonary function of adult patients with asthma or with asthma-chronic obstructive pulmonary disease (COPD) overlap syndrome [79]. The mechanisms of the toxicity of PM2.5 towards the respiratory system have been investigated. These studies show that the environmental particle itself acts as a chronic inflammatory agent due to its low clearance rate and high deposition efficiency. In addition, the PM2.5 surface is rich in metals including ferrous iron, copper, zinc and manganese, as well as in polycyclic aromatic hydrocarbons and lipopolysaccharide, which are derived from power generation, industrial activity and biomass burning. These components can induce an inflammatory reaction [76]. An epidemiological study indicated that each 10 μg/m^3^ increase in PM2.5 was associated with a 19–30% increase in lung cancer mortality (Table 1) [80]. Considering the cross-border nature of airborne particles, international efforts to improve air quality are needed.

Air pollutants also originate from domestic heating and cooking with poor ventilation [16]. Cigarette smoke is another common air pollutant, as well as a foreign body. Smoking is the primary risk factor for COPD, which is characterized by chronic lung inflammation [81]. The presence of COPD is associated with six-times the risk for the development of lung cancer compared to smokers without COPD, indicating that COPD is an independent risk factor for lung cancer (Table 1) [82].

## 3. Animal Models for Inflammation-Related Cancer Chemoprevention Studies

Chemoprevention is the use of pharmacological or natural agents that inhibit or delay the development of cancer [83]. Various animal models that resemble human inflammation-related cancers have been previously generated by genetic engineering or by bacterial/chemical induction, and cancer prevention research has been facilitated by the use of those models (Table 2). We review these animal models in this section.

### 3.1. Esophageal Cancer

The rat model for esophago-duodenal anastomosis is known to sequentially progress from reflux esophagitis to Barrett’s esophagus and then to esophageal adenocarcinoma within 50 weeks of the operation [84]. Mouse reflux models yield a lower incidence of adenocarcinoma (7%) compared to rat models (40%) [95,96,97]. The rat reflux model is therefore widely used for the exploration of chemopreventive agents.

### 3.2. Gastric Cancer

Transgenic mice that overexpressed human gastrin and were infected with *Helicobacter pylori* (*H. pylori*) uniformly developed gastric adenocarcinoma by 24 weeks [98]. However, there have been no descriptions of non-genetically engineered mice that have developed gastric adenocarcinoma, which is probably a reflection of poor host adaptation to *H. pylori* [99]. *Helicobacter felis* (*H. felis*) isolated from the feline stomach can colonize the murine stomach similar to *H. pylori* and sequentially induce chronic gastritis, atrophy, intestinal metaplasia and adenocarcinoma [99,100]. However, unlike *H. pylori* infection of humans, neutrophil infiltration is less prominent in *H. felis*-induced murine gastritis, and *H. felis* is deficient in the production of the *Helicobacter* cytotoxin, vacA and the pro-inflammatory cytokine inducer, cagA [99,101]. Mice infected with *H. pylori* have a low susceptibility to gastric carcinogenesis even when a chemical carcinogen is used [102]. Besides these mouse models, a Mongolian gerbil was successfully established to mimic human *H. pylori* infection and chronic inflammation, in which the bacteria were detectable throughout the one-year study period [100]. Gastric adenocarcinomas that are very similar to those in humans were developed in 64% of *H. pylori*-infected Mongolian gerbils treated with *N*-methyl-*N*′-nitro-*N*-nitrosoguanidine at Week 50 [85].

### 3.3. Colon Cancer

Oral administration of dextran sulfate sodium (DSS) is well known to induce colitis in animals. DSS causes defects in epithelial barrier integrity, thereby enhancing colonic mucosal permeability to allow the entry of luminal antigens and bacteria into the mucosa, resulting in an inflammatory response [103]. Repeated administration of DSS that mimics acute and chronic phases of human ulcerative colitis induces chronic inflammation that is characterized by severe tissue injury of both the lamina propria and submucosa [103,104,105]. The use of DSS in combination with intraperitoneal injection of azoxymethane (AOM), a chemical carcinogen, results in 100% incidence of colonic tumors, whereas the incidence is only 13% to 19% when DSS is administered alone [86]. The incidence of neoplasia is also increased by administration of DSS in combination with other carcinogens, such as dimethylhydrazine (DMH) or 2-amino-1-methyl-6-phenylimidazo[4,5-b] pyridine [86,87].

Genetically-modified animal models of colon cancer have been generated. For example, the *Apc*^Min/+^ mouse carries a germline mutation that converts codon 850 of the murine *Adenomatous polyposis coli* (*Apc*) gene from a leucine to a stop codon [106] and that mimics the development of adenomatous polyps in humans with familial adenomatous polyposis (FAP). However, the most common sites of tumors of *Apc*^Min/+^ mice is the small intestine [87]. *Apc*^Min/+^ mice exhibited adenomas in the small intestine at the age of five weeks [107] and subsequently developed intestinal adenomas (100% incidence). In the colon, precancerous lesions such as aberrant crypt foci or β-catenin accumulated crypts are observed, but the incidence of adenocarcinoma is no more than about 20% [108]. DSS administration to *Apc*^Min/+^ mice leads to colonic adenocarcinoma formation in all cases [87,108]. Since *Apc*^Min/+^ mice are *Apc* gene hetero-deficient, they are already in the initiated phase of tumor development. Therefore, DSS-induced inflammation acts as a promoter for colonic adenocarcinoma development [87].

### 3.4. Hepatocellular Carcinoma

Reliable methods to induce chronic inflammation-related hepatocellular carcinoma (HCC) in rodents are the use of chemicals or of transgenic approaches.

Hepatitis B or C viruses (HBV or HCV) can infect human hepatocytes subsequently leading to chronic inflammation and HCC development. In contrast to humans, mice are resistant to infection with HBV and HCV [109]. Transgenic mice carrying the full HBV genome except for the core protein were initially developed to model chronic HBV infection; however, HCC did not develop [110]. After this first report in 1985, transgenic mice overexpressing the HBV surface antigen in hepatocytes were established. This model exhibits chronic inflammation with necrosis, which inevitably leads to HCC [88].

Fourteen kinds of transgenic mice carrying HCV genes, such as the HCV polyprotein, and core protein alone or in combination with envelope proteins have been previously generated [109]. However, these HCV infection models either developed HCC without inflammation or did not form carcinomas [111]. Considering that there are no mouse models for hepatitis C-associated chronic inflammation-induced HCC, HBV transgenic mice are suitable as a mouse model that mimics the chronic carrier state of cancer-prone hepatitis virus infection.

Chemical carcinogens are also widely used to initiate hepatocarcinogenesis in animals. Diethylnitrosamine (DEN) was found to induce HCC in rodents in 1966 [112]. DEN is converted into a DNA alkylating agent by cytochrome P450 of hepatocytes and acts as a complete carcinogen if intraperitoneally injected into two-week-old mice [109]. The metabolic activation of DEN also generates ROS [109]. However, single injection of DEN results in carcinoma formation without cirrhosis. Therefore, the pathological process of the DEN-elicited rodent HCC is different from that of human HCC. In 2005, a rat model of DEN-induced liver injury that reproduces the sequence of cirrhosis and HCC that is observed in humans was established [89]. Once-a-week intraperitoneal injection of DEN for 16 weeks causes cirrhosis and multifocal HCC in all rats, similar to the case in human HCC [89].

Intraperitoneal injection of carbon tetrachloride (CCl_4_) induces pericentral necrosis of hepatocytes and inflammatory cell infiltration. In CCl_4_ treatment alone, only 25% of mice showed HCC [90]. In contrast, HCC was found in 50% of mice when a single injection of DEN, functioning as a tumor initiator, was followed by repeat treatment with CCl_4_, used as a tumor promoter, for 14 weeks.

### 3.5. Cholangiocarcinoma

Syrian golden hamsters infested with the liver fluke, *Opisthorchis viverrini* (*O. viverrini*), have been used as a model for cholangiocarcinoma. Infestation of the liver fluke alone rarely leads to cholangiocarcinoma. However, 100% incidence of bile duct cancers resembling those seen in humans resulted from the infestation prior to administration of *N*-nitrosodimethylamine (NDMA) [91]. The effect of liver fluke infestation and NDMA dose on the development of bile duct cancer is synergistic [113], indicating that there are several mechanisms underlying infestation-related carcinogenesis [114]. Firstly, the presence of the parasite mechanically damages bile duct epithelial cells that have a mutation that is caused by the carcinogen, resulting in increased cell proliferation, which fixes the DNA mutation [115,116]. Secondary, ROS and nitric oxide (NO) released by inflammatory cells cause DNA damage [114,117]. The third possibility is that inflammatory cells produce pro-inflammatory cytokines [114]. A fourth possible explanation is that *O. viverrini* secretes exosomes, one kind of membrane vesicle containing proteins, mRNA, miRNAs and DNAs [118], to promote cholangiocyte proliferation and IL-6 production [119].

### 3.6. Biliary Tract Cancer

Pancreaticobiliary maljunction (PBM) is characterized by abnormal fusion of the pancreatic and biliary ducts [120]. A PBM model was developed using the Syrian golden hamster [121]. Cholecystoduodenostomy in hamsters causes reflux of pancreatic juice into the biliary tract; as a result, pancreatic enzymes and secondary bile acid induce chronic inflammation with injury to biliary epithelia [122]. Biliary tract cancer developed in 41% to 82% of *N*-nitrosobis(2-oxopropyl)amine subcutaneously-injected hamsters after cholecystoduodenostomy [92].

### 3.7. Pancreatic Ductal Adenocarcinoma

Approximately 90% of human pancreatic ductal adenocarcinomas (PDAC) harbor mutations in codon 12, 13 or 61 of the K-*ras* gene [123,124], suggesting that K-*ras* is a driver gene in PDAC. However, only 50% of transgenic mice carrying a mutation of codon 12 of the K-*ras* allele (K-*ras*-mutated mice) developed PDAC [93]. When caerulein, an inducer of pancreatitis, was intraperitoneally-injected into K-*ras*-mutated mice constantly for six months, all of the mice had PDAC [93]. This result shows that chronic pancreatitis is necessary for the induction of PDAC and that K-*ras* mutation alone is insufficient for pancreatic carcinogenesis.

### 3.8. Skin Cancer

Two-stage skin carcinogenesis was developed in the 1940s. In the first stage, initiation occurs following a single administration of 7,12-dimethylbenz[a]-anthracene (DMBA). In the second stage, benign papillomas and/or invasive squamous cell carcinomas (SCC) developed by repeated treatment with 12-*O*-tetradecanoylphorbol-13-acetate (TPA), an inflammatory agent, to the initiated skin [94]. The DMBA/TPA skin model is used for screening of cancer chemopreventive compounds [125]. DMBA generates a point mutation in Ha*-ras*. TPA stimulates inflammation and the proliferation of Ha*-ras*-mutated cells [94]. Papillomas developed in about 80% of the mice by 22 weeks after initiation; the frequency of conversion of papilloma to carcinoma was about 20% at Week 32 [126]. A whole-exome sequencing study showed that 18% to 44% of the genes in DMBA/TPA-induced SCC, including Ha*-ras*, K*-ras* and *p53*, overlapped with genes in human SCC [127]. The DMBA/TPA skin tumor model therefore mimics human skin carcinogenesis at the genetic level.

### 3.9. Experimental Models of Foreign Body-Induced Carcinogenesis

In addition to infection, administration of a chemical substance or implantation of a foreign body also induces inflammation-related carcinogenesis. The first experimental evidence for a foreign body-induced tumor was reported in 1941 [71]. Most animal models of foreign body-induced tumorigenesis do not require a chemical carcinogen.

For example, 79% heterozygous *p53*-deficient (*p53*^+/−^) mice developed spontaneous sarcomas via induction of *p53* loss of heterozygosity at a mean time of 35 weeks after a piece of plastic plate (1 mm × 5 mm × 10 mm, polystyrene, used as a culture dish) was subcutaneously implanted [128]. Thus, an inflammatory reaction against a foreign body is sufficient for tumorigenesis. The carcinogenic potential of a foreign body depends on its properties [71]. Solid, smooth and large foreign bodies are more potent inducers of chronic inflammation than more roughened, smoothened and smaller ones [129]. As examples of foreign body-induced tumors, human or rodent immortalized cell lines that had been implanted attached to a plastic plate or a glass bead into mice or rats grew progressively in 8% to 100% of animals regardless of the origin of the cell (species, epithelial or non-epithelial cells) [71]. Another approach to establish this model is by using regressive tumors or precancerous cells.

FPCK-1-1 cells that are derived from a colonic polyp of a patient with FAP are non-tumorigenic when injected subcutaneously into nude mice. However, when these cells were attached to a piece of plastic plate and implanted into a subcutaneous space, the cells spontaneously converted into progressively-growing, moderately-differentiated adenocarcinoma cells in 65% of the mice [130]. The plastic plate initially induces acute inflammation, which then transitions to chronic inflammation [130]. A highly proliferative fibrous stroma composed mainly of fibroblasts was formed 120 days after plastic plate implantation. When FPCK-1-1 cells were injected into stromal tissues that were surrounded by a plastic plate, they converted into adenocarcinoma cells [130]. This result showed that the malignant conversion of FPCK-1-1 cells occurred not due to the plastic plate itself, but due to the plastic plate-induced fibrous stroma. NO derived from a chronically-inflamed lesion caused the conversion of FPCK-1-1 cells [131]. Moreover, the actin-filament bundling protein fascin-1 was found to be a suppressor of anoikis (apoptotic cell death as a consequence of insufficient cell-to-substrate interactions) and to drive the malignant conversion of FPCK-1-1 cells [132]. This malignant conversion seldom occurs in adenoma cells in the presence of a gelatin sponge, which is spontaneously absorbed in a short period and thus induces only the early phase of inflammation, indicating that the conversion requires chronic inflammation [130]. It should be noted that the carcinogenic inflammation was not induced in colon tissue, which is an orthotopic site for colon carcinogenesis, but in a subcutaneous space, which is an ectopic site. This evidence indicates that causes or sites of inflammation do not account for colon carcinogenesis, but that long-standing inflammation is necessary for colon carcinogenesis [130].

We have introduced chronic inflammation as a common cause of inflammation-related cancers in this review. However, acute inflammation also induces tumor formation experimentally. QR-32 cells (a mouse fibrosarcoma clone) regressed spontaneously after injection into syngeneic C57BL/6 mice, but could grow indefinitely in vitro [133]. Subcutaneous implantation of a gelatin sponge (3 mm × 5 mm × 10 mm) induces inflammatory cell (mainly neutrophils) infiltration. As mentioned above, the sponge is naturally absorbed about four weeks after implantation, and therefore, transition from acute to chronic inflammation is unlikely to occur when using a sponge [71]. The regressive QR-32 cells become tumorigenic after implantation into a pre-inserted piece of sponge. Moreover, the sponge-infiltrated inflammatory cells convert QR-32 cells into tumorigenic cells when both cells are mixed and injected subcutaneously [133]. Elimination of neutrophils by administration of an anti-neutrophil antibody inhibited the acquisition of malignant phenotype by QR-32 cells [134]. These findings show that neutrophil infiltration is needed for inflammation-related carcinogenesis [133,134]. There are advantages in using a gelatin sponge for investigating inflammation-related carcinogenesis. Since sponge-infiltrated inflammatory cells can be collected by treating the sponge with collagenase, it is possible to quantify the number of infiltrated cells, determine the cell types and analyze the molecular expression profiles of the inflammatory reaction [135].

## 4. Ten Mechanisms Involved in Inflammation-Related Carcinogenesis-Based Chemoprevention

Cancer prevention is the ultimate goal of inflammation-related carcinogenesis research. Chemoprevention research by using animal models of inflammation-related carcinogenesis as described above started in the late 1990s and continues to this day.

**Figure 3:**
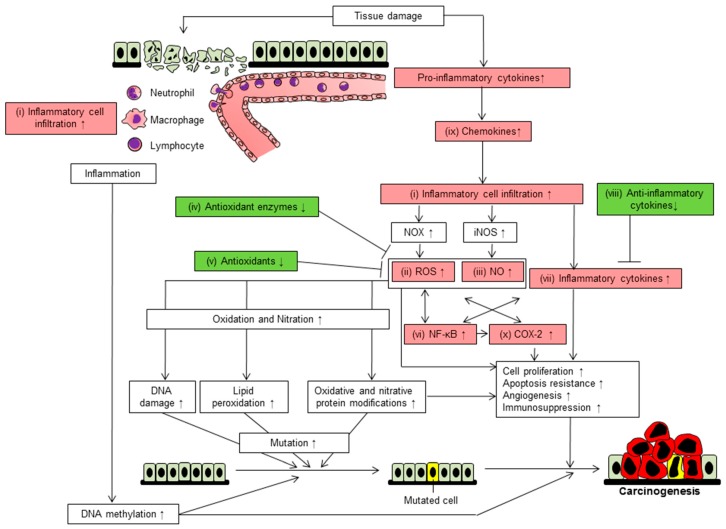
Schematic mechanism of inflammation-induced cancer development. Tissue damage causes inflammatory cell infiltration (i). Leukocytes produce ROS (ii) and NO (iii) resulting in oxidative/nitrative stress (DNA damage, lipid peroxidation, protein modification and, thus, mutation). Reduction of antioxidant enzymes (iv) and antioxidants (v), which scavenge ROS, leads to enhancement of oxidative stress. A positive feedback loop between NF-κB (vi) and pro-inflammatory cytokines (vii) is necessary for inflammation to become chronic. Anti-inflammatory cytokines (viii) are downregulated in inflammation-related carcinogenesis. Chemokines (ix) recruit leukocytes into inflammatory sites. In addition to ROS, NO and pro-inflammatory cytokines, COX-2 (x) promotes cell proliferation and angiogenesis and suppresses apoptosis and immunosurveillance. Inflammation also causes DNA methylation, which results in aberrant gene expression. Ten possible chemopreventive targets are shown in the red boxes. Factors that are decreased are shown in the green boxes. Pointed arrows indicate promotion/activation while T-shaped arrows indicate suppression.

Chemopreventive agents act through a combination of various mechanisms. By the study of these mechanisms of action, we summarized 10 mechanisms that are involved in the promotion of inflammation-related cancer development. These mechanisms are: (i) inflammatory cell infiltration; (ii) ROS; (iii) NO; (iv) reduction of antioxidant enzymes; (v) reduction of antioxidants; (vi) activation of NF-κB; (vii) upregulation of pro-inflammatory cytokines; (viii) downregulation of anti-inflammatory cytokines; (ix) elevation of chemokines; and (x) induction of cyclooxygenase (COX)-2 (Figure 3).

### 4.1. Inflammatory Cell Infiltration

Tissue injury caused by factors such as infection or a foreign body induces the sequential infiltration of neutrophils and monocytes (Figure 3). Granulocyte macrophage colony-stimulating factor released from epithelial cells or fibroblasts induces the differentiation of monocytes into M1 macrophages [136]. IL-4 works with macrophage colony-stimulating factor to induce to M2 macrophage polarization [137]. Tumor-associated macrophages (M2-like macrophages) promote inflammation-related carcinogenesis [138]. Infiltrated (activated) neutrophils, but not circulating or bone marrow neutrophils, are involved in carcinogenesis [133,134]. Depletion of macrophages using clodronate inhibited macrophage infiltration, resulting in suppression of AOM/DSS-induced mouse colon carcinogenesis [139]. Therefore, not only neutrophils, but also macrophages are necessary for cancer development in chronic inflammatory conditions. Indeed, the number of myeloperoxidase-positive cells (neutrophils and macrophages) was higher in the colonic mucosa of patients with inflammatory bowel disease (IBD) or its associated cancer than in normal mucosa [140], suggesting that inflammatory cell infiltration also plays a key role in human carcinogenesis.

Chemokines and adhesion molecules function in the recruitment of neutrophils and monocyte into inflammatory sites [141]. Integrin β2 is the key adhesion molecule for neutrophil extravasation. C-C motif chemokine receptor (CCR)2 is a specific receptor for the monocyte-tropic chemokine, C-C motif chemokine ligand (CCL)2. Genetic deletion of integrin β2 or CCR2 inhibited neutrophil/monocyte infiltration and protected mice from inflammation-related carcinogenesis [134,142]. Thus, inhibition of the initial process of inflammation, i.e., the infiltration of inflammatory cells, is a target for the prevention of chronic inflammation and carcinogenesis (Table 3).

### 4.2. Reactive Oxygen Species

Oxidative stress can lead to mutations and increased cell proliferation, and therefore, it plays a crucial role in inflammation-related carcinogenesis.

High ROS accumulation results in oxidative damage to DNA, protein or lipids, while a small increase in ROS acts as a growth signaling molecule in both normal and cancer cells [212]. Moreover, ROS is mutagenic across species [213]. In acute inflammation, the infiltrated inflammatory cells generate a massive amount of ROS to kill the invading pathogens [214,215]. If the acute inflammatory response fails to eliminate the pathogens and the inflammatory process persists, the sustained overproduction of ROS induces DNA damage and the proliferation of normal cells, which are associated with an increased risk of neoplastic transformation [214].

The bactericidal function of phagocytes including neutrophils depends on the generation of superoxide from the NADPH oxidase complex, which consists of cytosolic proteins (gp40*^phox^*, gp47*^phox^*, gp67*^phox^* and Rac) and a membrane-bound complex carrying cytochrome b_558_ (gp91*^phox^*, the catalytic core of phagocyte NADPH oxidase and gp22*^phox^*) [216,217]. In gp91*^phox^*^−/−^ mice, inflammation-related tumor development and metastasis were suppressed. Adoptively-transferred wild-type-derived infiltrated phagocytes into gp91*^phox^*^−/−^ mice recovered the acquisition of tumorigenicity and metastatic potential [218].

ROS further generates other reactive species (e.g., malondialdehydes (MDA) and 4-hydroxynonenal (4-HNE)) through lipid peroxidation. MDA and 4-HNE induce point mutation of the proto-oncogene K-*ras* and the tumor suppresser gene *p53* (Figure 3), thereby acting as a driving force for malignancy in chronic pancreatitis and IBD [219].

### 4.3. Nitric Oxide

NO is also released from infiltrated cells in chronic inflammatory tissues and causes alterations in DNA. NO is involved in colon cancer [220] and esophageal cancer [221] associated with inflammation. The main mechanisms of ROS and NO in inflammation-related carcinogenesis are DNA base modifications and strand breaks resulting in DNA-replication errors and genomic instability (Figure 3) [214]. There are at least two mechanism of NO-mediated carcinogenesis. First, NO converts colonic adenoma cells to adenocarcinoma cells by inducing the acquisition of resistance to anoikis [131]. Second, NO inactivates DNA repair enzymes and p53 proteins via post-translational modifications, such as nitrosylation, nitration and deamination (Figure 3) [222].

### 4.4. Reduction of Antioxidant Enzymes

The ROS level is determined by the rates of both ROS production and of ROS scavenging [212]. Therefore, suppression of the ROS production system or promotion of ROS scavenging activity is an effective strategy to prevent carcinogenesis.

In an experimental inflammation-related tumorigenesis model, an inverse correlation was observed between the frequency of inflammatory cell-induced somatic mutation or tumor formation and the activity of intracellular antioxidant enzymes (manganese superoxide dismutase (Mn-SOD) and glutathione peroxidase) [223]. Moreover, treatment with polysaccharide K [177] or an orally-available SOD [176] suppressed inflammation-related tumorigenesis by increasing Mn-SOD via induction of inflammatory cytokines.

### 4.5. Reduction of Antioxidant

Free radicals have an unpaired electron. Antioxidant vitamins C and E donate an electron to a free radical, thereby scavenging it. These antioxidant vitamins inhibit lipid peroxidation and nitration of tyrosine residues of proteins [224,225,226,227]. An epidemiological study showed that high intakes of vitamins C and E exhibited inverse associations with gastric cancer in *H. pylori*-infected subjects compared with non-infected individuals [228]. γ-Tocopherol, a major form of vitamin E, when present at 0.1% in the diet decreased the number of adenomatous polyps by 85% in the AOM/DSS colon cancer model [179]. Thus, the preventive effect of antioxidants on inflammation-related carcinogenesis has been observed both in human studies and in animal experiments.

### 4.6. Activation of NF-κB

NF-κB (a heterodimer of p50/NF-κB1 and p65/RelA) is found in the cytoplasm where it is bound to IκBs that prevent its activation in unstimulated cells. IκB phosphorylation causes its ubiquitin-proteasomal degradation, leading to the release of NF-κB, which then enters the nucleus and functions as a transcription factor of inflammation-related genes [229].

NF-κB has been found to be constitutively activated in inflammatory diseases, such as IBD and COPD [230,231]. Its activation is induced by pro-inflammatory cytokines (tumor necrosis factor (TNF)-α, IL-1β, IL-6 and IL-8), ROS, bacterial infection and ultraviolet irradiation [229,232]. NF-κB promotes the transcription of pro-inflammatory cytokines, leukocyte chemoattractant proteins (chemokine (C-X-C motif) ligand (CXCL)12, CCL2 and CCL3), COX-2 and endothelial adhesion molecules (E-selectin, vascular cell adhesion molecule 1 and intercellular adhesion molecule 1), leading to enhancement of inflammatory cell infiltration and inflammatory reactions [232,233]. NF-κB activation also increases the expression of ROS-producing enzymes (gp91^phox^, xanthine oxidase) or inducible NO synthase (Figure 3), resulting in the promotion of cell proliferation, the acquisition of apoptosis resistance and induction of genetic instability [214,234,235].

A recent report showed that NF-κB promoted TNF-α secretion, which, in turn, activated more NF-κB, in acute myeloid leukemia [236]. This NF-κB/TNF-α positive feedback loop also exists in inflammation associated with Barrett’s carcinogenesis [237], indicating that it is a common mechanism in both epithelium and non-epithelium. Inflammation-related cancer development may be suppressed by any one of the inhibitions of NF-κB activation, downregulation of pro-inflammatory cytokines or upregulation of an anti-inflammatory cytokine (IL-10) due to breakdown of the NF-κB/TNF-α positive feedback loop.

### 4.7. Upregulation of Pro-Inflammatory Cytokines

Pro-inflammatory cytokines (e.g., IL-1β, IL-6 and TNF-α) are produced by macrophages, B and T lymphocytes, endothelial cells and fibroblasts. These cytokines exert paracrine and autocrine effects via binding to their transmembrane receptors [238,239,240]. These cytokines are involved in the promotion of cell proliferation, induction of angiogenesis, autophagy and inhibition of apoptosis [238]. In the DMBA/TPA skin tumor model, 100% of wild-type mice had tumors (7.3 tumors per mouse). In contrast, only 38% of TNF-α-null mice developed tumors (0.9 tumors per mouse) because keratinocyte hyperproliferation and inflammation were diminished by deletion of TNF-α [241].

TNF-α and interferon-γ induce autophagy, a cellular degradation process involving the amino acid recycling for cellular survival and proliferation [160,242]. Melatonin prevents the development of adenocarcinoma by suppressing of autophagy in DMH/DSS colon cancer model [160].

The inflammasome is a multi-protein complex functioning as a platform for the activation of caspase-1, which then lead to the maturation of IL-1β and IL-18 [243,244]. The activation of the inflammasome in immune cells (dendritic cells and macrophages) increases the recruitment of suppressive immune cells, such as myeloid-derived suppressor cells and regulatory T cells and facilitates angiogenesis through the release of fibroblast growth factor-2 and vascular endothelial growth factor [245].

Epidermal growth factor (EGF) is secreted by platelets and macrophages [246], and its expression is increased in inflammatory diseases and at wound sites [247,248]. To examine the effect of EGF on the tumor progression of weakly-tumorigenic and nonmetastatic rat mammary adenocarcinoma (ER-1) cells, the cells were exposed to EGF (100 ng/mL) for a short (24 h) or a long (one month) period in vitro [249]. Each EGF treatment period converted ER-1 cells into tumorigenic and metastatic cells. Their malignant features were reversible during the short exposure to EGF, but the acquired malignant phenotypes were fixed by long exposure. The acquisition of malignant phenotypes was prevented by the addition of an antioxidant, *N*-acetylcysteine or selenium [182,249]. It is therefore assumed that EGF that is present in an inflammatory environment stimulates ROS production, resulting in oxidative DNA damage and malignant conversion.

### 4.8. Downregulation of Anti-Inflammatory Cytokines

Anti-inflammatory cytokines such as IL-10 are produced by CD8^+^ T cells [250]. IL-10 inhibits NF-κB signaling at two levels: (i) through blocking of the activity of IκB kinases and (ii) through inhibition of NF-κB DNA binding [251]. All IL-10-deficient mice spontaneously developed colitis at the age of nine weeks. In 10 to 31-week-old mice, the incidence of colorectal adenocarcinomas reached 65% [252]. IL-10 has anti-inflammatory and then anti-tumorigenic properties, since it suppress levels of IL-6 and TNF-α [239].

### 4.9. Elevation of Chemokines

Chemokines recruit leukocytes into inflammatory sites. A high serum level of CXCL13, a B-cell chemoattractant, was associated with poor prognosis, bone marrow invasion and the presence of Epstein-Barr virus DNA in non-Hodgkin lymphoma patients [253]. In addition to CXCL13, the expression level of CCL2, a monocyte chemoattractant, was 30- to 50-times higher in the colonic mucosa from patients with ulcerative colitis and Crohn’s disease than in that from controls [254]. CCL2 overexpression was also observed in the AOM/DSS colitis-associated carcinoma model [142]. The enhanced intracolonic macrophage infiltration and tumor development in this model were suppressed by using mice deficient in the CCL2-specific receptor, CCR2 [142]. Inhibition of chemokines decreases inflammatory cell infiltration and eventually attenuates carcinogenesis.

### 4.10. Induction of Cyclooxygenase-2

Prostaglandin E (PGE)2 is synthesized in multiple-steps: first, arachidonic acid is released from membrane-bound phospholipids by phospholipase A2; next, arachidonic acid conversion to prostaglandin H2 is mediated by COX; finally, PGE2 is produced by PGE synthase [255,256]. PGE2 causes increased cell proliferation, inhibition of apoptosis, stimulation of angiogenesis and immunosuppression in various cancers (Figure 3) [257]. In 1897, Hoffmann synthesized aspirin, a nonsteroidal anti-inflammatory drug (NSAID). Vane was the first to show that the active mechanism of aspirin was that of an inhibitor of COX [258]. The IARC evaluates NSAIDs, such as aspirin and sulindac, as cancer chemopreventive agents [259]. A clinical trial in the United Kingdom indicated that the use of aspirin for about five years was effective in the prevention of colon cancer [260]. In addition to colon cancer, a chemopreventive effect of aspirin and other NSAIDs has also been reported for esophageal, gastric, lung, breast and prostate cancers [261]. COX-2 is induced by an inflammatory stimulus (infection, a foreign body, alcohol or tobacco), whereas COX-1 is constitutively expressed in gastrointestinal epithelium, renal tubules and platelets [229,239,262]. The NSAIDs aspirin, diclofenac, ibuprofen, indomethacin, naproxen and piroxicam are nonselective inhibitors of COX isozymes, and therefore, they increase the risk of gastrointestinal events, including bleeding and ulcer [263,264]. Shortly after the first of those reports, selective COX-2 inhibitors (celecoxib, etodolac, meloxicam, rofecoxib) were developed in order to reduce adverse effects [263]. A case-control study suggests that NSAIDs including celecoxib and rofecoxib might reduce the risk of patients with Barrett’s esophagus developing esophageal adenocarcinoma [265].

Selective and nonselective COX-2 inhibitors (MF-Tricyclic and sulindac, respectively) lower PGE2 levels and inhibit esophagitis and the development of adenocarcinoma in a rat model of Barrett’s esophagus [211]. This cancer preventive effect was also shown in an *H. pylori*-infected gastric cancer model, the AOM/DSS-induced colon cancer model and a pancreatic cancer model using caerulein and K-*ras* mutated mice [192,210,266]. Besides NSAIDs, fermented brown rice, rice bran with *Aspergillus oryzae* [184] and methanol extracts from the fruit of *A. communis* and the leaf of *A. communis* [147] also prevent inflammation-related carcinogenesis of the colon or skin by decreasing COX-2 expression (Table 3).

## 5. Candidate Chemopreventive Agents against Inflammation-Related Carcinogenesis

Table 3 presents a summary of 79 candidate chemopreventive agents reported in 70 primary journal articles using the above-described animal models of inflammation-related carcinogenesis. The information sources for this review include PubMed (from 1996 to 2017, Available online: https://www.ncbi.nlm.nih.gov/pubmed).

**Figure 4:**
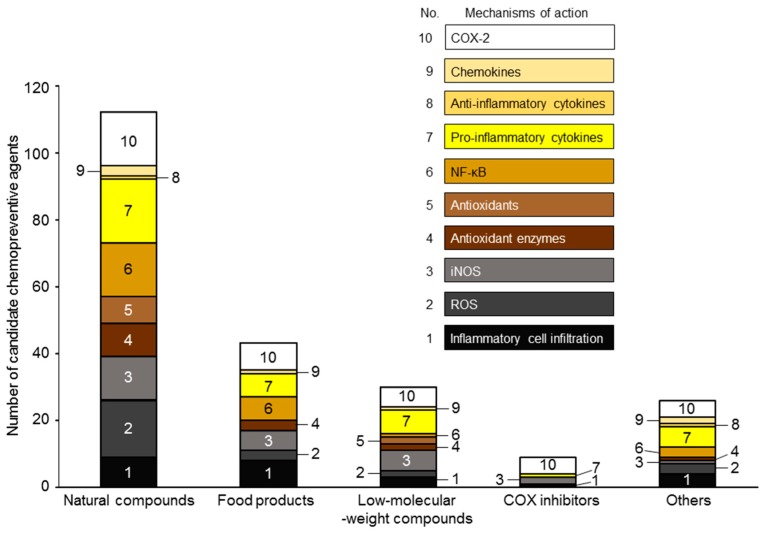
Natural compounds and food products have multiple chemopreventive mechanisms of action against inflammation-related carcinogenesis. The numbers of mechanisms of action of natural compounds, food products, low-molecular weight compounds, COX inhibitors and others against inflammation-related cancer development were calculated based on Table 3.

These 79 agents are classified into five groups: 34 natural compounds; 16 food products; 14 low-molecular-weight compounds; 5 COX inhibitors; and 10 others. The first four groups account for 87% of the total number of isolated agents. The mechanisms of action of these groups are listed in Figure 4 and are classified into the ten above-described mechanisms involved in inflammation-related carcinogenesis. Natural compounds followed by food products have the highest number of mechanisms of action. In contrast, low-molecular-weight compounds and COX inhibitors have a much lower number of mechanisms of action. These findings indicate that natural compounds and food products prevent inflammation-related carcinogenesis more effectively than low-molecular-weight compounds and specific molecular-targeted inhibitors. Of note, food products are low-cost because they are not perceived as “medicine”, and they are safe for long-term administration [267,268]. Cancer cases/deaths due to infection (inflammation) are expected to increase rapidly in low-income and middle-income countries within the next few decades [269]. Therefore, food products with anticarcinogenic/antiphlogistic effects may be ideal for cancer prevention in those countries.

## 6. Future Prospects

Chronic inflammation is central and common to the pathogenesis of not only carcinogenesis, but also cardiovascular disorders (arteriosclerosis, polyarteritis nodosa, aortitis syndrome and myocarditis), autoimmune diseases (systemic lupus erythematosus, rheumatoid arthritis, Crohn’s disease, type 1 diabetes, Hashimoto’s thyroiditis, Graves’ disease and sarcopenia), metabolic disorders (metabolic syndrome, type 2 diabetes and obesity) and neurological diseases (Alzheimer’s dementia, Parkinson’s disease and depression) [270,271,272,273,274,275,276,277,278,279,280,281,282,283]. Centenarians who are older than 100 years have higher levels of C-reactive protein, a sensitive indicator of inflammation, than younger people, indicating that chronic inflammation is also associated with healthy life expectancy [284]. The natural compounds and food products with preventive effects against inflammation-related cancers that are summarized in this review are expected to inhibit the above-listed inflammatory diseases because these agents have multiple inhibitory mechanisms of action.

Figure 1 shows that inflammation-related cancers develop in most organs/tissues. On the other hand, some inflammatory diseases do not increase cancer risk [285]; there has been no report showing that psoriasis or rheumatoid arthritis induces inflammation-related carcinogenesis. We assume two possible hypotheses for the difference in carcinogenic property between inflammatory diseases: (i) particular organs/tissues have resistance to carcinogenesis; (ii) the susceptibility of organs/tissues to carcinogenesis depends on the quality or the degree of the inflammatory reaction. Elucidation of these issues will lead to further understanding of the mechanism of inflammation-related carcinogenesis.

## Figures and Tables

**Table 1:** Cause-and-effect relationship between inflammation and its associated carcinogenesis in humans.

Sites of Inflammation-Related Carcinogenesis	Causes of Inflammation/Pathological Condition		
	Definitely Carcinogenic Agents (Group 1)	Presumed Carcinogenic Agents (Group 2A to 3 and the Others)	References
Eye	HIV type 1		[8]
		UV-associated skin inflammation	[8]
Lip		UV-associated skin inflammation	[8]
Oral cavity	HPV type 16		[8]
		HPV type 18	[8]
		Gingivitis	[9]
		Lichen planus	[9]
		Leukoplakia	[10]
		Periodontitis	[11]
Salivary gland		Sialadenitis	[9]
Tongue		HPV	[12]
		Caries	[13]
Tonsil	HPV type 16		[8,12]
Nasopharynx	EBV		[8,10,12]
Pharynx	HPV type 16		[8]
		Asbestos	[8]
Oropharynx		HPV	[12]
Larynx	Asbestos		[8]
		HPV type 16	[8]
Thyroid		Chronic lymphocytic thyroiditis	[14]
		Hashimoto’s thyroiditis	[14]
Esophagus		Gastric reflux, esophagitis	[9,10]
		Barrett’s esophagus	[10]
		Barrett’s metaplasia	[9]
		*Neisseria mucosa*	[15]
		*Neisseria sicca*	[15]
		*Neisseria subflava*	[15]
Lung	Asbestos		[8]
	Coal gasification		[8]
	Outdoor air pollution		[8,10,16]
	Tobacco smoke/smoking		[8,10]
		Asthma	[17]
		Bronchitis	[9]
		COPD	[18]
		Interstitial pneumonia	[19]
		Sarcoidosis	[20]
		Silicosis	[9]
		Tuberculosis	[21]
		*Chlamydia pneumoniae*	[22]
		HPV type 16	[23]
		HIV type 1	[24]
Lung mesothelium	Asbestos		[8,10]
		Silicosis	[25]
Breast		HERV-K	[26]
		Inflammatory breast cancer	[10]
Stomach	*Helicobacter pylori*		[8,10,12]
		Asbestos	[8]
		EBV	[8,10]
		Chronic atrophic gastritis	[10]
Liver	HBV		[8,10,12]
	HCV		[8,10,12]
	*Clonorchis sinensis*		[8,10]
	*Opisthorchis viverrini*		[8,10]
		Cirrhosis	[10]
		HDV	[27]
		HIV type 1	[8]
		*Schistosoma japonicum*	[8,10]
		Hemochromatosis	[28]
		α-1-anti-trypsin deficiency	[28]
		Alcohol	[28]
Bile duct	*Clonorchis sinensis*		[12]
	*Opisthorchis viverrini*		[12]
		Primary sclerosing cholangitis	[29]
		Bile acids-associated cholangitis	[9]
Gall bladder		Gall bladder stone-associated cholecystitis	[9,10]
		Primary sclerosing cholangitis	[29]
		Pancreaticobiliary maljunction	[30]
		*Salmonella typhimurium*	[10]
		*Salmonella enterica serovar* Typhi	[31]
Pancreas		Chronic pancreatitis	[10]
		Alcoholism-associated pancreatitis	[9]
		Hereditary pancreatitis	[32]
		Alcohol	[33]
Colon and Rectum		Bile acids-associated coloproctitis	[9]
		Inflammatory bowel diseases	[9,10,34]
		Cytomegalovirus	[35]
		EBV	[35]
		HPV	[35]
		JCV	[35]
		*Bacteroides*	[35]
		*Clostridium septicum*	[36]
		*Escherichia coli*	[35]
		*Helicobacter pylori*	[35]
		*Streptococcus bovis*	[35]
		*Streptococcus gallolyticus*	[37]
		*Schistosoma japonicum*	[8,10]
		Asbestos	[8]
Bladder	*Schistosoma haematobium*		[8,10,12,38]
		Cystitis	[10]
		Urinary catheter-associated cystitis	[9,39]
Anus	HIV type 1		[8]
	HPV type 16		[8]
		HPV types 18, 33	[8]
		Anal fistula	[40]
Testis		EBV	[41]
Prostate		Prostatitis	[42]
		Proliferative inflammatory atrophy	[10]
		Gonorrhea	[43]
		*Trichomonas vaginalis*	[44]
Ovary	Asbestos		[8]
		Pelvic inflammatory disease	[9]
		Endometriosis	[45]
Uterine cervix	HPV types 16, 18, 31, 33, 35, 39, 45, 51, 52, 56, 58, 59		[8]
	HIV type 1		[8]
		HPV types 26, 53, 66, 67, 68, 70, 73, 82	[8]
		Herpes simplex virus	[10]
Penis	HPV type 16		[8]
		HIV types 1	[8]
		HPV types 18	[8]
Vulva	HPV type 16		[8]
		HIV types 1	[8]
		HPV types 18, 33	[8]
		Lichen sclerosis	[9,46]
Vagina	HPV type 16		[8]
		HIV types 1	[8]
Skin	UV-associated skin inflammation		[8,10]
		Chronic osteomyelitis	[47]
		HIV types 1	[8]
		HPV types 5, 8	[8]
		MCV	[48]
Melanoma		UV-associated skin inflammation	[9]
Non-melanomatous skin cancer		Cutaneous HPV types	[48]
Central nerve		JCV	[49]
Endothelium (Kaposi’s sarcoma)	HIV type 1		[8,10]
	KSHV		[8]
Vasculature		*Bartonella*	[50]
Hodgkin’s lymphoma		EBV	[12]
		HIV type 1	[51]
Non-Hodgkin lymphoma		EBV	[12]
		HBV	[52]
		HCV	[12]
		HTLV-1	[12]
Lymphoma	EBV		[8,10]
	HCV		[8]
	HIV type 1		[8]
	HTLV-1		[8,10]
	KSHV		[8]
		HIV type 2	[53]
		Hashimoto’s thyroiditis	[9]
		Sjögren’s syndrome	[9]
		Childhood celiac disease	[54]
		HBV	[55]
		HTLV-1	[56]
		Malaria	[10]
Orbital lymphoma		*Chlamydia psittaci*	[57]
Thyroid lymphoma		Hashimoto’s thyroiditis	[58]
Lymphoma in the pleural cavity		EBV	[59]
Pyothorax-associated lymphoma		EBV	[60]
MALT lymphoma	*Helicobacter pylori*		[8,12]
Small-bowel lymphoma		*Campylobacter jejuni*	[61]
Cutaneous lymphoma		*Borrelia burgdorferi*	[62]
DLBC lymphoma		*Helicobacter pylori*	[12]
Adult T-cell leukemia	ATL (HTLV-1)		[63]
T-cell lymphoma		EBV	[64]
Burkitt’s lymphoma	EBV		[65]
B-cell lymphoma		EBV	[66]
Primary effusion lymphoma		KSHV	[67]

ATL, adult T-cell leukemia; COPD, chronic obstructive pulmonary disease; DLBC, diffuse large B-cell; EBV, Epstein-Barr virus; HBV, hepatitis B virus; HCV, hepatitis C virus; HDV, hepatitis D virus; HERV-K, human endogenous retrovirus type K; HIV, human immunodeficiency virus; HPV, human papillomavirus; HTLV-1, human T-cell lymphotropic virus type 1; JCV, JC virus; KSHV, Kaposi sarcoma herpes virus; MALT, mucosa-associated lymphoid tissue; MCV, *Molluscum contagiosum* virus; UV, ultraviolet.

**Table 2:** Animal models for inflammation-related carcinogenesis aimed at the development of chemoprevention.

Treatment	Carcinogen	Animal	Arising Tumor	Reference
Esophagojejunostomy	None	Rat	Esophageal adenocarcinoma	[84]
*H. pylori* infection	MNNG	Mongolian gerbil	Gastric adenocarcinoma	[85]
DSS	None	Mouse	Colorectal adenocarcinoma	[86]
DSS	AOM	Mouse	Colorectal adenocarcinoma	[86]
DSS	DMH	Mouse	Colorectal adenocarcinoma	[87]
DSS	PhIP	Mouse	Colorectal adenocarcinoma	[86]
DSS	None	*Apc*^Min/+^ mouse	Colorectal adenocarcinoma	[87]
None	None	HBV-transgenic mouse	Hepatocellular carcinoma	[88]
None	DEN	Rat	Hepatocellular carcinoma	[89]
CCl_4_	DEN	Mouse	Hepatocellular carcinoma	[90]
*O. viverrini* infection	NDMA	Hamster	Cholangiocarcinoma	[91]
Choledochojejunostomy	*N*-nitrosobis(2-oxopropyl)amine	Hamster	Biliary carcinoma	[92]
Caerulein	None	K-*ras* mutated mouse	Pancreatic ductal adenocarcinoma	[93]
TPA	DMBA	Mouse	Squamous cell carcinoma	[94]

AOM, Azoxymethane; *Apc*, *adenomatous polyposis coli*; CCl_4_, carbon tetra chloride; DEN, diethylnitrosamine; DMBA, 7,12-dimethylbenz[a]-anthracene; DMH, dimethylhydrazine; DSS, dextran sulfate sodium; HBV, hepatitis B virus; *H. pylori*, *Helicobacter pylori*; MNNG, *N*-methyl-*N*′-nitro-*N*-nitrosoguanidine; NDMA, *N*-nitrosodimethylamine; *O. viverrini*, *Opisthorchis viverrini*; PhIP, 2-amino-1-methyl-6-phenylimidazo[4,5-b] pyridine; TPA, 12-*O*-tetradecanoylphorbol-13-acetate.

**Table 3:** Chemopreventive agents against the 10 possible mechanisms of inflammation-related carcinogenesis.

Prevention Strategy	Chemopreventive Agent [Reference]	Type of Agent
**I. Inhibition of inflammatory cell infiltration**	Apocynin [143], apple oligogalactan [144], FBRA [145], *Ganoderma lucidum* [146], MEFA [147], MELA [147], PAG [148], γ-TmT [149]	Food product
	Auraptene [150], canolol [151], genistein-27 [152], geraniol [153], inotilone [154], micheliolide [155], nobiletin [156], tumerone [150], vitamin D [157]	Natural compound
	Hexaphosphate inositol [158], inositol [158], statin hydroxamate [159]	Low-molecular weight compound
	Melatonin [160]	Amino acid and its derivative
	Sulindac [161]	COX inhibitor
	Cholera-toxin [162]	Protein
	Oligonucleotides [163]	Oligonucleotides
	13-HOA [164]	Fatty acid
**II. Inhibition of ROS**	Juzen-taiho-to [165], oligonol [166], protandim [167]	Food product
	Auraptene [150], benzyl isothiocyanate [168], caffeine [169], crocin [170], DBM [171], digitoflavone [172], geraniol [153], GOFA/β-CD [173], menthol [174], organomagnesium [175], oxykine [176], PEITC [171], PSK [177], silibinin [178], tumerone [150], vitamin E [179], 3,3-diindolylmethane [180]	Natural compound
	Bismuth subnitrate [165], 3-aroylmethylene-2,3,6,7-tetrahydro-1H-pyrazino[2,1-a]isoquinolin-4(11bH)-ones [181]	Low-molecular weight compound
	Melatonin [160], *N*-acetylcysteine [182], selenium [182]	Amino acid and its derivative
**III. Suppression of iNOS**	EVOO [183], FBRA [184], MEFA [147], MELA [147], oligonol [166], PAG [148]	Food product
	Astaxanthin [185], baicalein [186], betaine [187], canolol [151], crocin [170], curcumin [188], inotilone [154], nobiletin [156], organomagnesium [175], pterostilbene [189], silibinin [178], UDCA [190], 5-OH-HxMF [191]	Natural compound
	Aminoguanidine [131], bezafibrate [192], GOFA-L-NAME [193], omeprazole [194], ONO-1714 [195], troglitazone [192]	Low-molecular weight compound
	Aspirin [196], nimesulide [192]	COX inhibitor
	Glutamine [197]	Amino acid and its derivative
**IV. Induction of antioxidant enzymes**	Juzen-taiho-to [165], oligonol [166], protandim [167]	Food product
	Crocin [170], DBM [171], digitoflavone [172], geraniol [153], GOFA/β-CD [173], menthol [174], organomagnesium [175], PEITC [171], PSK [177], 3,3-diindolylmethane [180]	Natural compound
	Bismuth subnitrate [165], 3-aroylmethylene-2,3,6,7-tetrahydro-1H-pyrazino[2,1-a]isoquinolin-4(11bH)-ones [181]	Low-molecular weight compound
	Melatonin [160]	Amino acid and its derivative
**V. Antioxidants**	Auraptene [150], benzyl isothiocyanate [168], caffeine [169], geraniol [153], oxykine [176], silibinin [178], tumerone [150], vitamin E [179]	Natural compound
	*N*-acetylcysteine [182], selenium [182]	Amino acid and its derivative
**VI. Inactivation of NF-κB**	Apple oligogalactan [144], EAPP [198], FBE [199], ME [199], oligonol [166], PAG [148], protandim [167]	Food product
	Astaxanthin [185], baicalein [186], betaine [187], crocin [170], curcumin [188], genistein-27 [152], GOFA/β-CD [173], inotilone [154], menthol [174], micheliolide [155], pterostilbene [189], silibinin [178], tricin [200], vitamin D [157], 3,3-diindolylmethane [180], 5-OH-HxMF [191]	Natural compound
	Cerulenin [201]	Low-molecular weight compound
	Glutamine [197], melatonin [160]	Amino acid and its derivative
	MiR-214 chemical inhibitor [202]	Oligonucleotides
**VII. Downregulation of pro-inflammatory cytokines**	Apple oligogalactan [144], EVOO [183], FBRA [145], *Ganoderma lucidum* [146], MEFA [147], MELA [147], oligonol [166]	Food product
	Astaxanthin [185], betaine [187], canolol [151], crocin [170], curcumin [188], digitoflavone [172], genistein-27 [152], GOFA/β-CD [173], isoliquiritigenin [139], micheliolide [155], organomagnesium [175], oroxylin A [203], pterostilbene [189], silibinin [178], tricin [200], triptolide [204], resveratrol [205], UDCA [190], vitamin D [157]	Natural compound
	Cerulenin [201], GOFA-L-NAME [193], NT1014 [206], omeprazole [194], statin hydroxamate [159], 3-aroylmethylene-2,3,6,7-tetrahydro-1H-pyrazino[2,1-a]isoquinolin-4(11bH)-ones [181], 5-aza-dC [207]	Low-molecular weight compound
	Glutamine [197], melatonin [160]	Amino acid and its derivative
	Aspirin [196]	COX inhibitor
	Cholera-toxin [162], α-lactalbumin [208]	Protein
	Oligonucleotides [163]	Oligonucleotides
	Eicosapentaenoic acid-free fatty acid [209]	Fatty acid
**VIII. Upregulation of anti-inflammatory cytokines**	PSK [177]	Natural compound
	Cholera-toxin [162]	Protein
**IX. Downregulation of chemokines**	FBRA [145]	Food product
	Auraptene [150], tumerone [150], vitamin D [157]	Natural compound
	Statin hydroxamate [159]	Low-molecular weight compound
	Glutamine [197]	Amino acid and its derivative
	Oligonucleotides [163]	Oligonucleotides
**X. Inhibition of COX-2**	EVOO [183], FBRA [184], *Ganoderma lucidum* [146], MEFA [147], MELA [147], oligonol [166], PAG [148], γ-TmT [149]	Food product
	Astaxanthin [185], betaine [187], canolol [151], crocin [170], curcumin [188], geraniol [153], inotilone [154], isoliquiritigenin [139], menthol [174], nobiletin [156], organomagnesium [175], pterostilbene [189], resveratrol [205], silibinin [178], 3,3-diindolylmethane [180], 5-OH-HxMF [191]	Natural compound
	Bezafibrate [192], cerulenin [201], GOFA-L-NAME [193], omeprazole [194], statin hydroxamate [159], troglitazone [192]	Low-molecular weight compound
	Glutamine [197], melatonin [160]	Amino acid and its derivative
	Aspirin [196], celecoxib [210], MF-tricyclic [211], nimesulide [192], sulindac [161]	COX inhibitor
	α-lactalbumin [208]	Protein
	Oligonucleotides [163]	Oligonucleotides
	Eicosapentaenoic acid-free fatty acid [209]	Fatty acid

COX-2, cyclooxygenase-2; DBM, dibenzoylmethane; EAPP, ethanol extracts from the aerial parts of *A. princeps* Pampanini cv. Sajabal; EVOO, extra virgin olive oil; FBE, fruiting body extract; FBRA, fermented brown rice and rice bran with *Aspergillus oryzae*; GOFA-L-NAME, 4′-geranyloxyferulic acid-*N*(omega)-nitro-l-arginine methyl ester; GOFA/β-CD, 3-(4′-geranyloxy-3′-methoxyphenyl)-2-trans propenoic acid/β-cyclodextrin; iNOS, inducible nitric oxide synthase; ME, mycelia extract; MEFA, methanol extracts of the fruit of *A. communis*; MELA, methanol extract of the leaf of *A. communis*; miR, microRNA; γ-TmT, γ-tocopherol-rich mixture of tocopherols; PAG, processed *Aloe vera* gel; PEITC, phenethyl isothiocyanate; PSK, polysaccharide K; ROS, reactive oxygen species; UDCA, ursodeoxycholic acid; 13-HOA, (±)-13-hydroxy-10-oxo-trans-11-octadecenoic acid; 5-OH-HxMF, 5-hydroxy-3,6,7,8,3′,4′-hexamethoxyflavone.

## Abbreviations

AD	Asian dust
AOM	Azoxymethane
*Apc*	*Adenomatous polyposis coli*
ATL	Adult T-cell leukemia
CCL	C-C motif chemokine ligand
CCl_4_	Carbon tetra chloride
COPD	Chronic obstructive pulmonary disease
COX	Cyclooxygenase
CXCL	Chemokine (C-X-C motif) ligand
DBM	Dibenzoylmethane
DEN	Diethylnitrosamine
DLBC	Diffuse large B-cell
DMBA	7,12-Dimethylbenz[a]-anthracene
DMH	Dimethylhydrazine
DSS	Dextran sulfate sodium
EAPP	Ethanol extracts from the aerial parts of *A. princeps* Pampanini cv. Sajabal
EBV	Epstein-Barr virus
EGF	Epidermal growth factor
EVOO	Extra virgin olive oil
FAP	Familial adenomatous polyposis
FBE	Fruiting body extract
FBRA	Fermented brown rice and rice bran with *Aspergillus oryzae*
GOFA/β-CD	3-(4′-Geranyloxy-3′-methoxyphenyl)-2-trans propenoic acid/β-cyclodextrin
GOFA-L-NAME	4′-Geranyloxyferulic acid-*N*(omega)-nitro-l-arginine methyl ester
*H. felis*	*Helicobacter felis*
*H. pylori*	*Helicobacter pylori*
HBV	Hepatitis B virus
HCC	Hepatocellular carcinoma
HCV	Hepatitis C virus
HDV	Hepatitis D virus
HERV-K	Human endogenous retrovirus type K
HIV	Human immunodeficiency virus
HPV	Human papillomavirus
HTLV-1	Human T-cell lymphotropic virus type 1
IARC	International Agency for Research on Cancer
IBD	Inflammatory bowel disease
IL	Interleukin
iNOS	Inducible nitric oxide synthase
JCV	JC virus
KSHV	Kaposi sarcoma herpes virus
MALT	Mucosa-associated lymphoid tissue
MCV	Molluscum contagiosum virus
MDA	Malondialdehydes
ME	Mycelia extract
MEFA	Methanol extracts of the fruit of *A. communis*
MELA	Methanol extract of the leaf of *A. communis*
MiR	MicroRNA
MNNG	*N*-Methyl-*N*′-nitro-*N*-nitrosoguanidine
Mn-SOD	Manganese superoxide dismutase
NDMA	*N*-Nitrosodimethylamine
NO	Nitric oxide
NSAID	Nonsteroidal anti-inflammatory drug
*O. viverrini*	*Opisthorchis viverrini*
PAG	Processed Aloe vera gel
PBM	Pancreaticobiliary maljunction
PDAC	Pancreatic ductal adenocarcinomas
PEITC	Phenethyl isothiocyanate
PGE	Prostaglandin E
PhIP	2-Amino-1-methyl-6-phenylimidazo[4,5-b] pyridine
PSK	Polysaccharide K
ROS	Reactive oxygen species
SCC	Squamous cell carcinoma
TNF	Tumor necrosis factor
TPA	12-*O*-Tetradecanoylphorbol-13-acetate
UDCA	Ursodeoxycholic acid
UV	Ultraviolet
γ-TmT	γ-Tocopherol-rich mixture of tocopherols
4-HNE	4-Hydroxynonenal
5-OH-HxMF	5-Hydroxy-3,6,7,8,3′,4′-hexamethoxyflavone
13-HOA	(±)-13-Hydroxy-10-oxo-trans-11-octadecenoic acid
